# Opiate Withdrawal Complicated by Tetany and Cardiac Arrest

**DOI:** 10.1155/2014/295401

**Published:** 2014-06-15

**Authors:** Irfanali R. Kugasia, Nehad Shabarek

**Affiliations:** Department of Internal Medicine, Lincoln Medical and Mental Health Center, 234 East 149th Street, Bronx, NY 10451, USA

## Abstract

Patients with symptoms of opiate withdrawal, after the administration of opiate antagonist by paramedics, are a common presentation in the emergency department of hospitals. Though most of opiate withdrawal symptoms are benign, rarely they can become life threatening. This case highlights how a benign opiate withdrawal symptom of hyperventilation led to severe respiratory alkalosis that degenerated into tetany and cardiac arrest. Though this patient was successfully resuscitated, it is imperative that severe withdrawal symptoms are timely identified and immediate steps are taken to prevent catastrophes. An easier way to reverse the severe opiate withdrawal symptom would be with either low dose methadone or partial opiate agonists like buprenorphine. However, if severe acid-base disorder is identified, it would be safer to electively intubate these patients for better control of their respiratory and acid-base status.

## 1. Introductions

Withdrawal from opiates is considered to be generally benign with the common symptoms being diaphoresis, piloerection, lacrimation, diarrhea, anxiousness, nonspecific abdominal and bodily pain sensation, and hyperventilation. It is rarely associated with life threatening complications, unlike withdrawal from benzodiazepine and alcohol which are frequently associated with life threatening complications and need close monitoring in an inpatient setting [1–3]. As a result, opiate antagonist like naloxone has been extensively and successfully used in the field and the emergency departments for diagnosing altered mental status from opiate overdose and reversing their complication of respiratory depression which is considered to be more life threatening [3–5]. However, in rare cases serious complications and fatal outcomes have been reported during opiate withdrawals, particularly in patients who are undernourished or have underlying electrolyte or cardiorespiratory abnormalities [3, 5, 6]. These complications considered to be either from direct toxic effects of naloxone or from increased catecholamine surge due to severe opiate withdrawal [4–6]. This case emphasizes the need for a cautious approach towards severe manifestations of simple opiate withdrawal symptom, like hyperventilation.

## 2. Case

**Figure 1:**
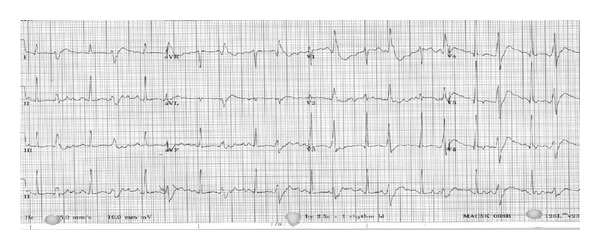
Initial EKG showing bigeminy—showing increased cardiac muscle excitability with origination of ventricular ectopics.

A 54-year-old female was brought to the hospital by EMT (emergency medical team) with suspicion of opiate overdose. EMT was called by a family member after the patient was found to be less responsive and difficult to arouse. The patient received 4 mg of naloxone subcutaneously in field by the EMT which improved patient's responsiveness but pushed her into severe opiate withdrawal. In the emergency department, the patient was found to be awake, alert, and anxious. She was not oriented but was able to follow simple commands. The patient was also diaphoretic, tachypneic with respiratory rate in 30 and tachycardic with heart rate in 150 s. Lungs were clear on auscultation with good bilateral air entry. Cardiovascualr exam was unremarkable with regular rhythm good pulses present in all four extremities. Abdominal exam was also unremarkable. Neuromuscular exam showed tremulousness and muscle fasciculation in different muscle groups of the face, upper limb, and lower extremities, exacerbated deep tendon reflex and normal motor strength with severe pain on movement of left hip. The patient had a past medical history of AIDS, hepatitis C, and bipolar disorder but as per her home medication list she was not on any treatment for them. At home, the patient was on 150 mg of methadone for maintenance and oral morphine sustained release and immediate release for pain control for her recent hip fracture. The first ABG done at the time of patient's ED presentation was on a nonrebreather mask; it showed pH 7.694, PCO_2_ 19.6 mmHg, PO_2_ 224 mmHg, O_2_ saturation of 100%, and lactate of 2.97 and her initial EKG showed bigeminy with the rest of the details obscured by artifacts as seen in Figure 1. In the next 30 minutes patient's respiratory rate had decreased to 18–25 breaths per minute at which time a repeat ABG was done on room air that showed pH of 7.641, PCO_2_ 20.3 mmHg, PO_2_ of 86 mmHg, and O_2_ saturation of 98% with lactate of 2.48. While being on telemetry, the patient had sinus tachycardia with PVCs and runs of bigeminy. The patient was subsequently deemed stable and taken for CT scan of heat and chest for further evaluation, while in CT room the patient went into generalized tetany and then into cardiac arrest with initial rhythm of ventricular tachycardia that degenerated to torsade de pointes and ventricular fibrillation. Code was run for 25 minutes, during which the patient was intubated and received 2 doses of epinephrine and 4 gm of magnesium and was shocked 3 times. She had a return of spontaneous circulation after the third shock. The patient was initially started on hypothermia protocol; however, it was abandoned as patient showed movement of extremities. The labs obtained prior to patient's cardiac arrest showed Na^+^ 138, K^+^ 3.4, Mg^+^ 2.0, Ca^2+^ 8.9, HCO_3_ 18, and anion gap of 16; however, this sample was hemolyzed. Repeat labs obtained 30 minutes after intubation showed K^+^ 3.5, Ca^2+^ 7.6, iCa^2+^ 3.92, Mg^+^ 2.6, and HCO_3_ 16 with anion gap of 17. EKG obtained after cardiac arrest showed sinus tachycardia. Urine toxicology obtained for the patient was positive only for methadone and opiates. Patient's serum albumin level was 2.9. The patient was extubated on day 2 and was discharged from hospital on day 8 of hospitalization.

## 3. Discussion

The most important concern after giving naloxone to a patient with opiate overdose is reversal of naloxone antagonism and patient slipping back into respiratory depression from opiate overdose. This is because the half-life of naloxone action is only 20–80 minutes, much shorter than many of the opiates [2, 3]. The initial impression in this case was that patient had respiratory alkalosis from her tachypnea due to acute opiate withdrawal and this was expected to reverse as quickly as the effect of naloxone wears off. However, repeat clinical evaluation and ABG after 30 minutes did not show any major change as the patient was still breathing at 25 breaths per minute and was tachycardic to 120–140 s. So other explanations for the clinical findings were sought. The consideration of pulmonary embolism was high on the differential as the patient had recent history of hip fracture and had an A-a gradient of almost 40 mmHg on the repeat arterial blood gas. As a result, decision was made to rule it out with a chest CT which came out to be negative for pulmonary embolism or any lung pathology. CT brain was also negative for any intracranial bleed.

**Figure 2:**
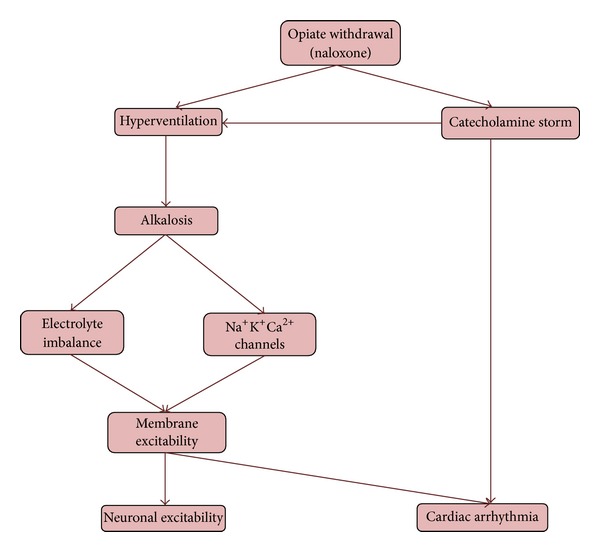
Mechanism explaining neuronal and cardiac excitability in opiate withdrawal.

The cardiac arrest observed in this case can be explained by three main reasons. Firstly, it could be from naloxone itself. Pulmonary edema and cardiac arrest from ventricular fibrillation have been reported with administration of naloxone. This was mostly seen in postoperative patient to whom naloxone was given to reverse the effect of opiates and in patient who was on high dose of opiate for pain relief who had some kind of cardiac comorbidity [6, 7]. These complications were considered to be from a sudden surge in catecholamine levels and have been mostly reported within minutes of IV naloxone administration [6, 7]. Since the cardiac arrest in this case occurred almost 1 hour after the naloxone which was given subcutaneously and negative chest CT for pulmonary edema, naloxone as the cause of cardiac arrest is less likely. Secondly, the cardiac arrhythmias observed in this patient like torsades de pointes and ventricular fibrillation are found in patient with prolonged QT interval. This is commonly seen with methadone overdose. The incidence of torsades de pointes with methadone is increased when methadone overdose is associated with electrolyte abnormality especially hypokalemia [8]. The cardiac arrest in this case could be easily attributed to methadone overdose associated with some electrolyte abnormalities. However, there was no conclusive evidence of prolonged QTc. The waves in initial EKG were obscured by artifacts and repeat EKG after successful resuscitation showed a QTc of 473. Also, none of this could explain the simultaneous occurrence of tetany and cardiac arrest observed in this case. Lastly, the cardiac arrhythmia and tetany could be explained by the severe alkalemia secondary to hypocapnia from patient's hyperventilation. As per patient's initial lab and ABG, she had severe primary respiratory alkalosis with mild high anion gap metabolic acidosis which was considered to be from patient's elevated lactate level from her tachypnea. Voluntary hyperventilation in normal healthy patients has been found to be associated with severe respiratory alkalosis, increase in anion gap from lactic acidosis, and significant change in serum K^+^ level [9, 10]. Severe alkalemia resulting from hyperventilation and hypocapnia has been shown to cause perioral numbness, cardiac arrhythmias, seizure, and tetany [11–13]. These complications can be either due to the direct effect of alkalemia on ion gated channels controlling membrane potential or secondary to its effect on serum potassium (K^+^) and ionized calcium (iCa^2+^) levels or from combination of both [14]. Putting together these pathophysiologic effects of severe alkalemia can result in neuronal and cardiac excitability. This can explain the initial findings of muscle fasciculation and bigeminy and later the tetany, polymorphic ventricular tachycardia, and ventricular fibrillation which were observed in this patient. A simplified schematic for this mechanism is depicted in Figure 2. Also notable was the rapid improvement in patient's clinical status and EKG finding after intubation with resolution of arrhythmia and overall good patient outcome.

To avoid severe opiate withdrawals and complications, a more conservative and cautious use of naloxone is advised. Intravenous route by well-trained personnel to better titrate the naloxone dose is recommended, as at low dose naloxone can reverse the respiratory depression from opiate overdose without causing overt withdrawal from opiates [3]. With subcutaneous and intramuscular administration of naloxone, the time to peak effect and peak effect are less predictable than intravascular administration. However, once severe withdrawal symptoms are identified, immediate treatment with low dose of methadone or partial opiate agonists like buprenorphine could help in reversing some of these severe withdrawal symptoms [2, 3]. Nonetheless, with concerns of respiratory depression from opiate overdose and risk of severe alkalosis from hyperventilation, early elective intubation and sedation should be highly considered, as these would provide better control of patient's respiratory system and in turn their acid-base status.
